# Inhibition of *Listeria monocytogenes* by Phage Lytic Enzymes Displayed on Tailored Bionanoparticles

**DOI:** 10.3390/foods11060854

**Published:** 2022-03-17

**Authors:** Edel Stone, Vincenzo Pennone, Kerri Reilly, Irene R. Grant, Katrina Campbell, Eric Altermann, Olivia McAuliffe

**Affiliations:** 1 Teagasc Food Research Centre, Moorepark, Fermoy, P61 C996 Cork, Ireland; edelmcstone@gmail.com (E.S.); vincenzopennone@hotmail.it (V.P.); 2 Institute for Global Food Security, School of Biological Sciences, Queens University, 19 Chlorine Gardens, BT9 5DL Belfast, Ireland; i.grant@qub.ac.uk (I.R.G.); katrina.campbell@qub.ac.uk (K.C.); 3 AgResearch Ltd., Palmerston North 4410, New Zealand; kerri.reilly@agresearch.co.nz (K.R.); eric.altermann@agresearch.co.nz (E.A.); 4 Riddet Institute, Massey University, Palmerston North 4442, New Zealand

**Keywords:** *Listeria monocytogenes*, bacteriophage, endolysin, amidase, bionanoparticles, BNPs

## Abstract

The high mortality rate associated with *Listeria monocytogenes* and its ability to adapt to the harsh conditions employed in food processing has ensured that this pathogen remains a serious problem in the ready-to-eat food sector. Bacteriophage-derived enzymes can be applied as biocontrol agents to target specific foodborne pathogens. We investigated the ability of a listeriophage endolysin and derivatives thereof, fused to polyhydroxyalkanoate bionanoparticles (PHA_BNPs), to lyse and inhibit the growth of *L. monocytogenes*. Turbidity reduction assays confirmed the lysis of *L. monocytogenes* cells at 37 °C upon addition of the tailored BNPs. The application of BNPs also resulted in the growth inhibition of *L. monocytogenes.* BNPs displaying only the amidase domain of the phage endolysin were more effective at inhibiting growth under laboratory conditions (37 °C, 3 × 10^7^ CFU/mL) than BNPs displaying the full-length endolysin (89% vs. 83% inhibition). Under conditions that better represent those found in food processing environments (22 °C, 1 × 10^3^ CFU/mL), BNPs displaying the full-length endolysin demonstrated a greater inhibitory effect compared to BNPs displaying only the amidase domain (61% vs. 54% inhibition). Our results demonstrate proof-of-concept that tailored BNPs displaying recombinant listeriophage enzymes are active inhibitors of *L. monocytogenes*.

## 1. Introduction

*Listeria monocytogenes* is a foodborne pathogen that is often associated with ready-to-eat food products such as deli meats, mixed salads, fresh dairy products and leafy greens [1,2]. If consumed in a contaminated food product, the organism can cause listeriosis; this is a rare but serious illness, particularly for at-risk groups including the young, the elderly and the immunocompromised [3]. The high mortality rate (20–30%) associated with the illness has resulted in stringent detection and control measures for *L. monocytogenes* in food processing environments. Despite these controls, the physiological resistance of the organism against low temperatures and high salt concentrations, and its ability to form biofilms, make this pathogen difficult to manage [4].

The use of bacteriophages (phages) as natural biocontrol agents against foodborne pathogens including *L. monocytogenes* has been investigated elsewhere [5,6]. As reported in these and other studies, the application of whole phages has been shown to significantly inhibit the growth of *L. monocytogenes* on different food matrices. Recombinant production of phage proteins, such as endolysins, is a useful alternative to the use of whole phages. Endolysins (lysins) are phage-encoded peptidoglycan hydrolases produced in phage-infected bacterial cells toward the end of the replication cycle [7]. Holins form membrane lesions so that lysins can reach the peptidoglycan and cleave the bacterial membrane, subsequently leading to host cell death and the release of newly formed phages into the environment [8]. Lysins acting against Gram-positive bacteria typically show a modular design, in which catalytic function and specific cell-wall recognition areseparated into two or more functional domains. Simplistically, lysins contain one N-terminal enzymatically active domain (EAD) and one C-terminal cell-wall-binding domain (CBD) [9]. The use of recombinant lysins allows the exploitation of phages that have a lysogenic life cycle and reduces the risk of the emergence of bacteriophage-insensitive mutants [9]. Lysins are also considered to be less host-specific and do not necessarily require actively growing host cells to bring about inhibition [10,11,12]. Previous work by our group demonstrated the inhibitory effect of the catalytic domain of the *L. monocytogenes* phage vB_LmoS_293 lysin on the formation of *L. monocytogenes* biofilms [13].

Polyhydroxyalkanoate bionanoparticles, or PHA_BNPs, have gained significant interest in a variety of applications in the biotechnology sector as an economically efficient, non-toxic, biodegradable method for the delivery of functional proteins and enzymes [14,15]. Polyhydroxyalkanoates (PHAs) are biopolyesters synthesized by cells in which they function as carbon reservoirs [16]. The enzyme PhaC permits protein fusions to both its C- and N-termini. As a result, the tailored BNPs can display proteins and enzymes on the surface in an orientated fashion without the enzymatic activity of the enzyme being lost [14]. PHA_BNPs offer distinct advantages over other possible expression methods. These include the covalent binding and stabilization of the protein in a uniform direction to the surface of the nanobead. The stabilizing matrix on the nanobeads enables ready deployment of proteins and enzymes in liquids or on surfaces, the expression of proteins in a one-step process, and the resulting high yield of product [17]. Effective uses of these BNPs have previously been demonstrated by Altermann et al. [14] wherein tailored BNPs lysed a range of rumen methanogen strains and reduced methane production by 97%. Similarly, Davies et al. [17] reported that tailored BNPs could act as a successful protective layer in PPE against Mycobacteria after a one log (91%) reduction was reported.

In this study, the hypothesis that PHA_BNPs can be successfully deployed as a potential production and delivery system for *L. monocytogenes*-specific phage-derived endolysins and their catalytic domains was validated. The objectives of this study were (1) to determine if PHA_BNPs displaying either lysin293 or amidase 293 can be produced in *E. coli* and subsequently purified; (2) to determine if assays can be developed to successfully measure the lytic activity of these proteins displayed on PHA_BNPs; (3) to determine if amidase293 will have equal or greater efficacy compared to lysin293 when displayed on PHA_BNPs; (4) to determine the effect of temperature on the activity of the PHA_BNPs; and (5) to determine if the concentration of bacterial cells (CFU/mL) has an effect on the activity of the PHA_BNPs. By meeting each of these objectives, this study would act as a proof-of-concept that these tailored BNPs could be exploited in the future as natural antimicrobials or sanitizing agents. Ultimately, two separate varieties of tailored BNPs were generated: the first variety, PHA_lysin293_BNPs, displayed the full-length lysin, lysin 293, of *L. monocytogenes* phage vB_LmoS_293; the second variety, PHA_amidase293_BNPs, displayed a truncated lysin harboring only the amidase domain of lysin 293, or amidase 293. The efficacy of these lysin-displaying BNPs against *L. monocytogenes* in both turbidity reduction assays and in growth inhibition experiments was tested to determine the potential of tailored BNPs as delivery mechanisms for phage-based biocontrol agents. 

## 2. Materials and Methods

### 2.1. Bacterial Strains, Plasmids and Culture Conditions

*L. monocytogenes* strain 473 (serotype 4e) was streaked from −80 °C stocks onto Tryptic Soy Agar (TSA; Becton Dickinson and Company, Le Pont-de-Claix, France) and incubated at 37 °C for 48 h. Actively growing *L. monocytogenes* cells were produced by selecting a single colony from these plates and inoculating this into 10 mL of Tryptic Soy Broth (TSB) and incubating for 18 h at 37 °C. *E. coli* BL21 (DE3) cells (Thermo Fisher Scientific, Dublin, Ireland) were grown in Lysogeny Broth (LB) liquid media (Neogen, Lancashire, UK) containing 50 μg/mL ampicillin (Amp; Merck Life Science Ltd., Wicklow, Ireland) and 64 μg/mL chloramphenicol (Cm; Merck Life Science Ltd., Wicklow, Ireland) at 37 °C with shaking. Table 1 lists the bacterial cells, plasmids and conditions used in this study. 

### 2.2. Bioinformatic Analysis of Phage vB_LmoS_293

The genome of phage vB_LmoS_293 has been previously sequenced and annotated, and is available in the GeneBank database with the Accession Number KP399678.1 [19]. The Basic Local Alignment Search Tool (BLAST) was used to analyze Open Reading Frame (ORF) 25 coding for lysin293, and the NCBI Conserved Domains Database [20] was used to identify the amidase domain [13].

### 2.3. Plasmid Construction for PHA BNP Generation 

The constructs used in this study were created according to Altermann et al. [14]. The PHA–BNP constructs used in this study were synthesized by GeneArt (Thermo Fisher Scientific, GENEART GmbH, Regensburg, Germany). The gene sequences used in this study can be found in Table S1. Briefly, the gene fusions of lysin293 and PhaC, and amidase293 and PhaC, were optimized for expression in *E. coli*. The synthetic gene was then incorporated into the pET14b vector under the control of the LacZ promoter. pET14b containing the PHA sequence only was also synthesized as a control (Table 1). Following synthesis, the pET14b plasmids were transformed into chemically competent *E. coli* BL21 (DE3) cells (Thermo Fisher Scientific, Dublin, Ireland) that contained the helper plasmid pMCS69, harboring the *phaA* and *phaB* genes required to synthesize PHA precursors [21]. pMCS69 was transformed into chemically competent *E. coli* DE3 cells. Briefly, 100 ng of DNA (pMCS69) was transformed into 50 μL of *E. coli* competent cells and incubated on ice for 30 min. The cells were heat-shocked at 42 °C for 60 s and placed on ice for 3 min. An amount of 500 μL of LB medium was added to the cells and incubated at 37 °C for 40 min with shaking. After incubation, 200 μL of the transformation mix was plated onto LB agar plates containing 50 μg/mL Cm. The plates were incubated at 37 °C overnight. Subsequently, the pET14b plasmids containing the gene fusions of PHA_lysin293, PHA_amidase293 or the PHA sequence only were transformed into competent *E. coli* BL21 (DE3) cells containing the helper plasmid pMCS69, following the method outlined above. Double transformants containing the pET14b plasmids and pMCS69 were plated onto LB agar plates containing 50 μg/mL Amp and 64 μg/mL Cm and incubated overnight at 37 °C. 

### 2.4. Generation of PHA-BNPs

PHA_BNPs were produced according to Altermann et al. [14]. Briefly, the transformants of interest were grown in 1 L of LB broth supplemented with 1% (*w*/*v*) glucose and with appropriate antibiotics (Amp (50 μg/mL), and Cm (64 μg/mL)) and at 37 °C with shaking (150 rpm). At an OD600 of 0.5, production of BNPs (PHA_lysin293_BNPs, PHA_amidase293_BNPs and PHA_BNPs) was induced by the addition of 1 mM Isopropyl β-D-1-thiogalactopyranoside (IPTG; Merck Life Science Ltd., Wicklow, Ireland). Following growth at 25 °C with agitation for 48 h, cells were harvested by centrifugation (6000× *g*, 5 min at 4 °C). Cell pellets were resuspended in 50 mM phosphate buffer with a pH of 7.5 and lysed via sonication (Vibracell Sonicator, Sonics and Materials, Newtown, CT, USA) on ice, with 20 s bursts at a medium intensity and 30 s rest intervals over a 10 min time interval. Recovery of BNPs was performed using ultracentrifugation at 21,000× *g* for 2 h at 4 °C in a Sorvall TH641 swing-out rotor (Thermofisher Scientific, Auckland, New Zealand) over a glycerol gradient, as described in [22]. After ultracentrifugation, the white band containing the PHA_BNPs at the glycerol gradient interface was extracted and brought to a volume of 45 mL using phosphate-buffered saline (PBS) (Life Technologies Ltd., Paisley, UK). The solution was centrifuged at 8000× *g* for 20 min to separate the purified PHA_BNPs from any remaining glycerol. After centrifugation, the supernatant was discarded and PHA BNP pellets were resuspended in phage buffer (10 mM Tris (pH 7.5), 10 mM MgSO4, 68 mM NaCl) at a concentration of 20 mg/mL with 20 μL/mL Tween 80 (Merck Life Science Ltd., Wicklow, Ireland). The purified PHA_BNPs were stored at −80 °C. When in use, the PHA_BNPs were stored at 4 °C and not continuously frozen and refrozen.

### 2.5. Lysis and Growth Inhibition Assays 

#### 2.5.1. Preparation of Bacterial Culture and Protein 

*L. monocytogenes* strain 473 (serotype 4e) was prepared following 18 h of incubation in TSB (Becton Dickinson and Company, Le Pont-de-Claix, France) at 37 °C under aerobic conditions. The concentrations of each of the PHA_BNPs, PHA_lysin293_BNPs and PHA_amidase293_BNPs, were adjusted to 0.25 mg/mL in PBS (Life Technologies Ltd., Paisley, UK). Protein concentration was confirmed with a Qubit protein quantification assay using the Qubit 4 Fluorometer (Invitrogen, Thermo Fisher, Singapore) following the manufacturer’s guidelines. Supplementary Figure S1 depicts the experimental design for the following assays.

#### 2.5.2. Application of PHA_BNPs for Lysis of *L. monocytogenes*

An amount of 100 µL TSB (Becton Dickinson and Company) was inoculated with approximately 1 × 10^7^ CFU/mL of *L. monocytogenes* strain 473, to which 0.25 mg/mL of PHA_lysin293_BNPs, PHA_amidase293_BNPs or control PHA_BNPs was added to give total reaction volumes of 200 μL in a 96-well plate. Samples were incubated at 37 °C, and the turbidity of the samples was measured at 30 min intervals for up to 3 h, by reading the absorbance of samples using a Synergy 2 BioTek 96-well-plate reader (BioTek Instruments, Inc., Winooski, VT, USA) at an OD of 600 nm. Optical densities were corrected according to Altermann et al. [14] using Equation (1).

Equation (1): Where n: sample taken at predefined time point; OD600 (n): corrected optical density at point n; OD600 (n)(a): measured optical density at point n; OD600 (0): measured optical density at time point 0; OD600 (n − 1): measured optical density at point n − 1; bc: test BNPs used; Lmc: *L. monocytogenes* control plus cells; bead: PHA_BNPs or PHA_lysin293_BNPs or PHA_amidase293_BNPs in the absence of *L. monocytogenes* cells.

(1) $${\mathrm{OD}}_{600\left(n\right)}={\;\mathrm{OD}}_{600\left(n\right)\left(a\right)}-\left({\mathrm{OD}}_{600\left(0\right)\left(\mathrm{bc}\right)}-{\;\mathrm{OD}}_{600\left(0\right)\left(\mathrm{Lmc}\right)}\right)+\left({\mathrm{OD}}_{600\left(n-1\right)\left(\mathrm{bead}\right)}-{\;\mathrm{OD}}_{600\left(n\right)\left(\mathrm{bead}\right)}\right)$$

#### 2.5.3. Application of PHA_BNPs for Growth Inhibition of *L. monocytogenes*

TSB was inoculated with approximately 1 × 10^7^ CFU/mL of *L. monocytogenes* strain 473, and 0.25 mg/mL of either PHA_lysin293_BNPs, PHA_amidase293_BNPs or the control PHA_BNPs was added for a total reaction volume of 200 μL. Samples were incubated at 37 °C and plated at 30 min intervals for up to 3 h on Listeria Chromogenic agar (Harlequin, Lancashire, UK). A total volume of 100 μL was taken and serially diluted, using Maximum Recovery Diluent (Oxoid Ltd., Basingstoke, UK), to a dilution of 10^−8^.The plates were incubated at 37 °C for 48 h. To assess the inhibitory nature of the beads at a lower starting cell number, TSB was inoculated with approximately 1 × 10^3^ CFU/mL of *L. monocytogenes* strain 473, and 0.25 mg/mL of either PHA_lysin293_BNPs, PHA_amidase293_BNPs or the control PHA_BNPs was added for a total reaction volume of 200 μL. Samples were incubated at 22 °C and plated at 30 min intervals over a 3 h period onto Listeria Chromogenic agar (Neogen, Lancashire, UK). The plates were incubated at 37 °C for 48 h. The percentage inhibition was calculated using CFU/mL data. 

### 2.6. Statistical Analysis 

Statistical analysis was performed using Prism Software GraphPad 9. A paired *t*-test was used for comparison between two groups. The data are presented as the standard error of mean (SEM) values. A *p*-value of 0.05 was considered statistically significant. The mean OD600 nm and standard deviations were calculated from two independent experiments with duplicates in each experiment.

## 3. Results

### 3.1. PHA_BNPs Displaying Lysin293 and Amidase293 Cause Lysis of L. monocytogenes 

To determine if the application of PHA_lysin293_BNPs and PHA_amidase293_BNPs result in the lysis of *L. monocytogenes* strain 473 (serotype 4e), turbidity reduction assays were conducted. The controls in these experiments consisted of cells of *L. monocytogenes* strain 473 in the absence of any PHA_BNPs (L. mono-PHA_BNPs) and cells of *L. monocytogenes* strain 473 in the presence of PHA_BNPs displaying no form of lysin (L. mono + PHA_BNPs). 

**Figure 1:**
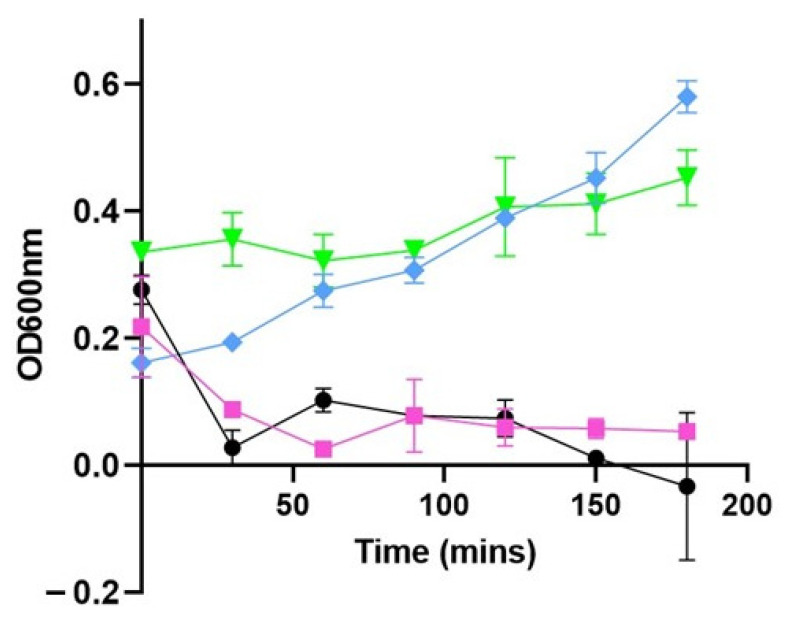
Experiment 1A: turbidity reduction assays performed at 37 °C using 1 × 10^7^ CFU/mL *L. monocytogenes* 473 (serotype 4e). The data have been adjusted according to Equation (1). *L. monocytogenes* strain 473 was inoculated into TSB containing PHA_lysin293_BNPs (pink symbols) (*n* = 4), PHA_amidase293_BNPs (black symbols) (*n* = 4), L. mono + PHA_BNP control (green symbols) (*n* = 4), and L. mono-PHA_BNPs (blue symbols) (*n* = 4). Absorbance at OD 600 nm was measured at 0, 30, 60, 90, 120, 150 and 180 min.

When applied at 37 °C to 1 × 10^7^ CFU/mL (OD 600 nm 0.2) of *L. monocytogenes* strain 473 (Experiment 1A), the addition of PHA_lysin293_BNPs resulted in a reduction in turbidity of 80% (*p* = 0.0126) and 76.71% (*p* = 0.0002) after 30 min, compared to the control without BNPs (L. mono-PHA_BNPs) and with BNPs without lysin (L. mono + PHA_BNPs), respectively (Figure 1). Under the same conditions, the application of PHA_amidase293_BNPs resulted in a reduction in turbidity of 81.5% (*p* = 0.0244) and 76.85% (*p* = 0.0012), compared to the control without BNPs (L. mono-PHA_BNPs) and with BNPs without lysin (L. mono + PHA_BNPs), respectively (Figure 1). In both cases, the reduction in optical density persisted throughout the duration of the assays, and the growth of *L. monocytogenes* strain 473 was inhibited for 3 h. 

### 3.2. PHA_BNPs Displaying Lysin293 and Amidase293 Cause Growth Inhibition of L. monocytogenes

**Figure 2:**
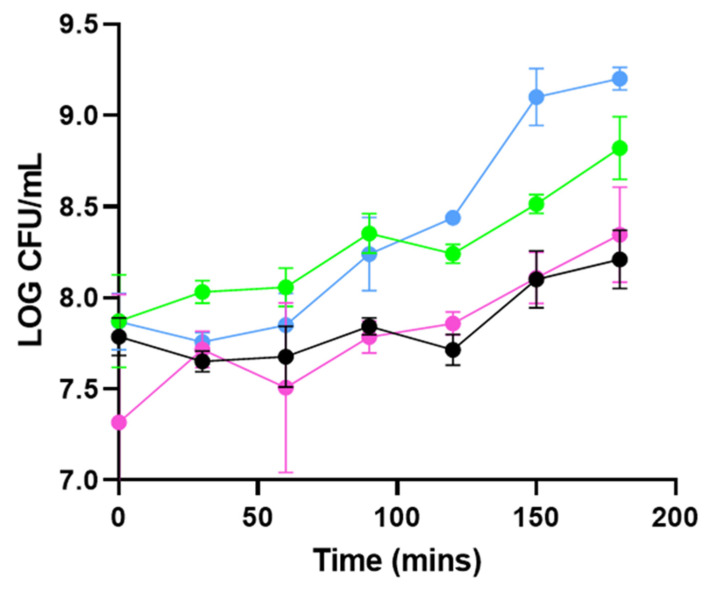
Experiment 1B: growth inhibition assays at 37 °C using 1 × 10^7^ CFU/mL *L. monocytogenes* 473 (serotype 4e). *L. monocytogenes* strain 473 was inoculated into TSB containing PHA_lysin293_BNPs (pink symbols) (*n* = 4), PHA_amidase293_BNPs (black symbols) (*n* = 4), L. mono + PHA_BNP control (green symbols) (*n* = 4), and L. mono-PHA_BNPs (blue symbols) (*n* = 4). Cells were incubated at 37 °C and samples taken for plating on Listeria Chromogenic Agar at 0, 30, 60, 90, 120, 150 and 180 min. The figure depicts total counts of *L. monocytogenes*.

To investigate the effects of PHA_lysin293_BNPs and of PHA_amidase293_BNPs on the growth of *L. monocytogenes* strain 473, cell counts (CFU/mL) were also determined. Two experiments were designed, one at 37 °C with a high starting inoculum (1 × 10^7^ CFU/mL; Experiment 1B), and one at 22 °C, with a starting inoculum that represents the concentration of *L. monocytogenes* commonly isolated from contaminated plants (1 × 10^3^ CFU/mL) (Experiment 2B) [23]. The controls in this group were similar to those used for the turbidity reduction assays. In experiment 1B (37 °C, 1 × 10^7^ CFU/mL), when compared to the cells-only control, the addition of PHA_lysin293_BNPs and PHA_amidase293_BNPs lowered the population numbers of *L. monocytogenes* by 84.4% (*p* = 0.008) and 89.5% (*p* = 0.0006), respectively, following 3 h of incubation (Figure 2). When compared to the L. mono + PHA_BNP control, the highest inhibition was seen at 3 h for PHA_amidase293_BNPs, which reduced the rate of growth by 75% (*p* = 0.0141) and 2 h for PHA_lysin293_BNPs (83% *p* = 0.0046). This experiment shows that these PHA_BNPs have no killing effect but have a slight inhibitory effect on the growth of *L. monocytogenes*. Compared to the L. mono + PHA_BNP control, the average inhibition over the course of 3 h was 66.5% (*p* = 0.0001) and 61.3% (*p* = 0.0002) when applying the PHA_amidase293_BNPs and PHA_lysin293_BNPs, respectively. When compared to the cells-only control the average inhibition over the course of 3 h was 83.1% (*p* = 0.0007) and 81.5% (*p* = 0.0008) when applying the PHA_amidase293_BNPs and PHA_lysin293_BNPs, respectively. Although there is slight inhibition shown for the duration of this experiment, there is significance shown between the controls and the test. 

**Figure 3:**
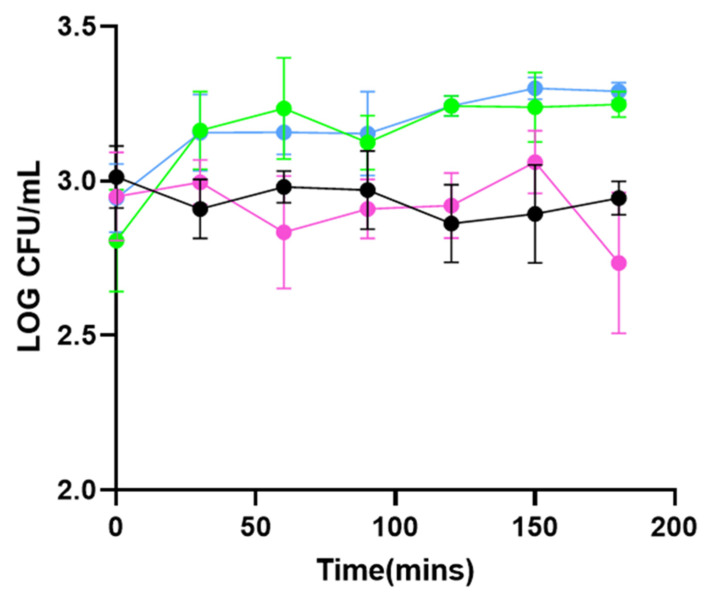
Experiment 2B: growth inhibition assays at 22 °C using 1 × 10^3^ CFU/mL *L. monocytogenes* 473 (serotype 4e). *L. monocytogenes* strain 473 was inoculated into TSB containing PHA_lysin293_BNPs (pink symbols) (*n* = 4), PHA_amidase293_BNPs (black symbols) (*n* = 4), L. mono + PHA_BNP control (green symbols) (*n* = 4), and L. mono-PHA_BNPs (blue symbols) (*n* = 4). Cells were incubated at 22 °C and samples taken for plating on Listeria Chromogenic Agar at 0, 30, 60, 90, 120, 150 and 180 min. The figure depicts total counts of *L. monocytogenes*.

In experiment 2B (22 °C, 1 × 10^3^ CFU/mL), the addition of the PHA_lysin293_BNPs and the PHA_amidase293_BNPs resulted in the inhibition of *L. monocytogenes* strain 473 by 61.5% (*p* = 0.0246) and 54.6% (*p* = 0.0111), respectively, compared to the L. mono-PHA_BNP control (Figure 3). The average inhibition exhibited upon addition of PHA_amidase293_BNPs over the 3 h period was 47.5% (*p* = 0.0025), and upon addition of PHA_lysin293_BNPs, was 46.7% (*p* = 0.0022). Like in experiment 1B, there is slight inhibition of *L. monocytogenes*. 

## 4. Discussion

This study investigated the potential for tailored PHA_BNPs (expressing a fusion of lysin293 or the amidase domain of this lysin) to lyse and inhibit the growth of *L. monocytogenes* cells in pure culture. Phage vB_LmoS_293, belonging to the family *Siphoviridae*, was previously isolated by our group from mushroom compost and was found to be specific for *L. monocytogenes* serotypes 4e and 4b [19,24]. An analysis of the genome of phage vB_LmoS_293 revealed that ORF 25 (nucleotide 19966–20916) encoded a 316-amino-acid endolysin (lysin293), belonging to the N-acetylmuramoyl-l-alanine amidase family (COG5632). BLASTp analysis revealed that the protein contained a PGRP element that functions in peptidoglycan recognition in the bacterial cell wall, as well as a catalytic domain (amidase293), belonging to the amidase 2 family (pfam015100) [13]. We have previously demonstrated the lytic capability of amidase293 on autoclaved cells of *L. monocytogenes* and its ability to inhibit the formation of an *L. monocytogenes* biofilm on stainless steel [13].

Both lysin293 and the amidase293 were successfully fused C-terminally to PhaC, which allowed the generation of PHA_BNPs. Two separate varieties of tailored BNPs weresuccessfully produced in *E. coli* and subsequently purified: the first displayed the lysin293 (PHA_lysin293_BNPs) and the second displayed the amidase293 (PHA_amidase293_BNPs). A series of assays were developed and optimized to determine the efficacy of these BNPs as lytic agents and/or growth inhibitors of *L. monocytogenes* (Supplementary Figure S2). 

The lytic ability of the BNPs were tested against *L. monocytogenes* strain 473, the host strain of phage vB_LmoS_293, in a series of turbidity reduction assays. At 37 °C, the application of both PHA_lysin293_BNPs and PHA_amidase293_BNPs resulted in a reduction in the turbidity of these test solutions. This reduction in turbidity is an indication that the application of these BNPs harboring the phage-derived enzymes results in the lysis of *L. monocytogenes* strain 473 cells. Interestingly, under these experimental conditions, it can be seen that amidase293 maintains the lytic ability of lysin293 when compared to the L. mono + PHA_BNP control. These turbidity-reduction assays also indicate that there is no significant difference between the rate of lysis when using amidase293 versus lysin293. With the lytic ability being maintained, and the rate of lysis not being hindered by truncating the lysin, it suggests that there is a level of substrate specificity in the N-terminal domain. Our group and others have made similar observations previously. CHAP_K_, the catalytic domain of the LysK endolysin from the *Staphylococcus aureus* phage, phage K, was as active, if not more active, than the full-length LysK [25]. We also reported that the host range of CHAP_K_ was broader than that of LysK [22]. More recently, Mayer et al. found that a truncated N-acetylmuramoyl-l-alanine amidase of a *Clostridium difficile* endolysin lysed cells of *C. difficile* faster than the full length lysin [23]. However, in this case, no increase in host range was observed with the truncated lysin. A host range comparison of lysin293 and amidase293 is an area that needs be further investigated.

Two experimental variables were altered in a subsequent experiment to better reflect the conditions in which *L. monocytogenes* would be found in the food processing environment. These conditions are a lower temperature (i.e., room temperature) and a lower concentration of cells (CFU/mL) that represents the levels of contamination that would generally be found in food-processing plants. When analyzing the growth kinetics of *L. monocytogenes* strain 473 in experiment 1B, the addition of PHA_lysin293_BNPs reduced the rate of growth of strain 473 by an additional 12.1% in comparison to PHA_amidase293_BNPs, although no significance was observed for this result (*p* = 0.986). This indicates that, under these experimental conditions, amidase293 retains the same lytic ability as lysin293 when displayed on PHA_BNPs. Interestingly, at 22 °C, the application of PHA_lysin293_BNPs and PHA_amidase293_BNPs resulted in the inhibition of *L. monocytogenes* strain 473, maintaining *L. monocytogenes* levels at approximately the same concentration as the starting inoculum over the course of incubation. As experiment 2B better represents the conditions of food-processing plants, it can be suggested that the application of PHA_lysin293_BNPs and PHA_amidase293_BNPs may result in an inhibition of *L. monocytogenes* in food-processing plants. 

Although the inhibition of *L. monocytogenes* in experiment 2B is markedly less than in the experiment 1B, there is an immediate decrease in the CFU/mL when the PHA_amidase293_BNPs are added in experiment 2B suggesting that under conditions where there is a lower starting inoculum and a lower temperature, the PHA_amidase293_BNPs not only inhibit the growth of *L. monocytogenes*, but reduce it (reduction in the concentration of the starting inoculum of *L. monocytogenes*) by up to 28.75% (120 min) (*p* = 0.002); however, a marginal (17.5% average) reduction in *L. monocytogenes* is seen throughout the entire 3 h timeline. A hypothesis as to why an inhibitory effect and no reduction are seen in experiment 1B may be due to the PHA_lysin293_BNP: *L. monocytogenes* ratio. In experiment B, the concentration of PHA_amidase/lysin293_BNPs per cell of *L. monocytogenes* is approximately 0.25 μg/mL (0.25 mg/mL/1 × 10^3^ CFU/mL); in experiment 1B, the concentration of PHA_amidase/lysin293_BNPs per cell of *L. monocytogenes* is 0.025 ng/mL (0.25 mg/mL/1 × 10^7^ CFU/mL). Thus, the ratio of PHA_amidase/lysin293_BNPs: cell of *L. monocytogenes* is 10,000 times greater in experiment 2B vs. experiment 1B. To achieve the same PHA_amidase/lysin293_BNP: cell of *L. monocytogenes* ratio in experiment 1B as in experiment 2B, a concentration of 2.5 mg/mL of proteins would be required. Additionally, a achieving the reduction of 17.5% seen in experiment 2B would mathematically mean adding 1.4 mg/mL of protein to achieve a result of 99.9% (3-log reduction). However, preliminary studies performed using varying concentrations of protein revealed that concentrations above 0.25 mg/mL increased the growth of *L. monocytogenes*. Changes in the storage buffer of the tailored PHA_BNPs may allow the use of higher concentrations of tailored PHA_BNPs and, inversely, lead to a greater decrease in *L. monocytogenes*. 

Other studies have been conducted using phage lysins linked to nanoparticles for the reduction of *L. monocytogenes*, but using autoclaved cells. Pennone described experiments similar to those outlined is this work, wherein PHA_amidase293_BNPs were applied to *L. monocytogenes* strain 473 that had been subjected to autoclaving (121 °C/15 min) [26]. Turbidity reduction assays showed a reduction of 33.9% and 38% when using 1 mg and 5 mg of PHA_amidase_BNPs, respectively [26]. In another report, Solanki et al. conjugated lysin Ply500 to silica nanoparticles and, when applied to iceberg lettuce, a 4-log reduction in *Listeria innocua* was observed [27]. 

These lysin PHA_BNPs are natural and decomposable, which is an advantage to chemical-based antimicrobials that may be applied in the food processing environment. The key findings from this research are that PHA_BNPs may act as a suitable delivery system of phage vB_LmoS_293 endolysin and amidase domains, maintaining the enzymes in a stable form and preserving their lytic ability without the use of any chaperone proteins for lysis.

The results show an initial proof-of-concept for the application of these tailored PHA_BNPs in the inhibition of *L. monocytogenes*. Future experiments will determine if the tailored PHA_BNPs can be applied to inhibit *L. monocytogenes* present on surfaces in food-processing plants in an approach similar to that used by Davies et al. 2021 [17], where Mycobacteriophage endolysins fused to biodegradable nanobeads were applied to solid surfaces (filter paper). As the PHA_BNPs are active in liquid suspensions, as indicated in this study, a potential option for their application includes spraying onto food-contact surfaces, as with traditional sanitizers. It is unlikely that these tailored BNPs will replace traditional sanitizers, but may act as an additional hurdle to controlling *L. monocytogenes* where this organism is particularly problematic. It should also be noted that the conditions tested in these sets of experiments are not reflective of the conditions found in food-processing plants. Although this study showed that a reduction in temperature (37 °C to 22 °C) and CFU/mL maintained the activity of these BNPs, future experiments will focus on the application of these BNPs at refrigeration temperatures, given the ability of *L. monocytogenes* to grow at 4 °C. Preliminary findings also indicate that the application of these tailored BNPs is time-limited, as they were shown to be ineffective when applied for more than 3 h. These findings suggest that the tailored BNPs may be ineffective when applied as an antimicrobial for long durations; however, they may be applied for sanitization purposes over shorter periods of time. Future experiments may focus on the optimization of cells to tailored PHA_BNP ratios, to determine if this inhibitory effect can be further increased. The effect of these BNPs on biofilms would also be an area of interest in the future, as Pennone et al. have reported that the amidase domain from this lysin inhibits *L. monocytogenes* biofilm formation on stainless steel surfaces. 

## 5. Conclusions

To summarize, the findings of this study show that when displayed on PHA_BNPs, the amidase domain of lysin293 exhibits the same lytic ability as the full-length lysin293 at both 22 °C and 37 °C. Preliminary results also indicate that the application of these tailored BNPs is time-limited, as they were shown to be ineffective when applied for longer than 3 h. 

The results are promising and show an initial proof-of-concept for the use of PHA_BNPs displaying listeriophage lysins as a potential biocontrol agent against *L. monocytogenes*. The production of these bionanoparticles does not entail any complex or expensive post-production processes. In this study, bacterial cells (*E. coli* DE3) produced PHA_BNPs in a one-step process that only requires simple disruption of the bacterial cells to free the PHA_BNPs. This holds promise for rendering future large-scale production of PHA_BNPs cost-effective. The application of these tailored BNPs was shown to be successful at both 37 °C and 22 °C, and at *L. monocytogenes* concentrations of approximately 1 × 10^7^ CFU/mL and 1 × 10^3^ CFU/mL. An advantage of using this technology over chemical-based sanitizers or chemical-inhibition techniques is that these BNPs are biodegradable and, therefore, could be released in the food processing plant and naturally degraded over time, thus posing no threat to human health. Further studies are required on an extensive strain set, at a larger scale and, ultimately, in food production environments to demonstrate the efficacy of tailored BNPs in food-production environments. The results obtained to date are encouraging, considering the potential future applications in food-processing plants where cross contamination of *L. monocytogenes* poses a major concern.

## Figures and Tables

**Table 1:** Plasmids used in this study, detailing insert, features, host and products.

Plasmid Name	Insert	Resistance	Host Bacterium	Product	Reference
pET14b-PHA_lysin293_BNPs	Gene fusion of lysin293 and PhaC	Amp^R^	*E. coli* BL21 (DE3)	PHA_lysin293_BNPs	This study
pET14b-PHA_amidase293_BNPs	Gene fusion of amidase293 and PhaC	Amp^R^	*E. coli* BL21 (DE3)	PHA_amidase293_BNPs	This study
pET14b-PHA_BNPs	PhaC sequence	Amp^R^	*E. coli* BL21 (DE3)	PHA_BNPs	This study
pMCS69 (helper plasmid)	N/A	Cm^R^	*E. coli* BL21 (DE3)	N/A	[18]

## Data Availability

The raw data supporting the conclusions of this article will be made available by the authors, without undue reservation, to any qualified researcher.

## Supplementary Materials

The following supporting information can be downloaded at: https://www.mdpi.com/article/10.3390/foods11060854/s1, Figure S1: pET-14b vector used in this experiment, Figure S2: Flow chart depicting the experimental design of the assays, Table S1: Gene sequences of lysin293 and the amidase domain, amidase293.
