# Supplementary material for: Inhibition of Listeria monocytogenes by Phage Lytic Enzymes Displayed on Tailored Bionanoparticles
Source: Foods. 2022 Mar 17;11(6):854. doi: 10.3390/foods11060854 (PMC8951524; doi:10.3390/foods11060854)
Supplement: Supplementary file 1 [file foods-11-00854-s001.zip › foods-1612798-supplementary.pdf]

Supplementary Materials

Table S1. Gene sequences of lysin293 and the amidase domain, amidase293.

>Endolysin 1 (Full length), ORF 25, phage vB_LmoS_293, Coordinates: 19966..20916<br>MSVLQYNYINKNQFSRPGYKLLRVSKIVMHYTANPGASADNHRRYFRDLKERYASAHIFIDDNEAICIIPLNEVAY<br>HANERSCKLTALQASTSYYRGGNANLTSIGIEMCLDKNWNITAATFNRSVDVAAELCKTYDLTPSDIIRHYDVTG<br>KNCPAPWVAKPSELTRFRNAVNAKLKGASQNKNRHDGKVVDSAPLLTKMDFKSSPFRMYKSGTEFLVYEHNQ<br>YWYKTYIDDKLYYMYKSFCDVVTKKDAKGRIKVRIKSAKDLRIPVWNNTKLSSGKIKWYAPNTKLAWYNNGKGY<br>LELWYPSDGWYYTANYFLKVDANSSPRHMATGKGAAASTQEGKSQPFKVTPGPFDPATWLEWSRQWQGTE<br>GNGHAAASGIPGLDALAGVKIAPAQLGDIQQRYMKDFSALWQAMAEGKAEATGPLHDRRFAGDAWRTNLPY<br>RFAAAFYLLNARALTELADAVEADAKTRQRIRFAISQWVDAMSPANFLATNPEAQRLLIESGGESLRAGVRNM<br>MEDLTRGKISQTDESAFEVGRNVAVTEGAVVFENEYFQLLQYKPLTDKVHARPLLMVPPCINKYYILDLQPESSL<br>VRHVVEQGHTVFLVSWRNPDASMAGSTWDDYIEHAAIRAIEVARDISGQDKINVLGFCVGGTIVSTALAVLAA<br>RGEHPAASVTLLTTLLDFADTGILDVFVDEGHVQLREATLGGGAGAPCALLRGLELANTFSFLRPNDLVWNYVV<br>DNYLKGNTPVPFDLLFWNGDATNLPGPWYCWYLRHTYLQNELKVPGKLTVCGVPVDLASIDVPTYIYGSREDHI<br>VPWTAAYASTALLANKLRFVLGASGHIAGVINPPAKNKRSHWTNDALPESPQQWLAGAIEHHGSWWPDWTA<br>WLAGQAGAKRAAPANYGNARYRAIEPAPGRYVKAKA
>Endolysin (Amidase Domain), ORF 25, phage vB_LmoS_293, Coordinates: 19966..20502<br>MSVLQYNYINKNQFSRPGYKLLRVSKIVMHYTANPGASADNHRRYFRDLKERYASAHIFIDDNEAICIIPLNEVAY<br>HANERSCKLTALQASTSYYRGGNANLTSIGIEMCLDKNWNITAATFNRSVDVAAELCKTYDLTPSDIIRHYDVTG<br>KNCPAPWVAKPSELTRFRNAVNAKLKGAVDANSSPRHMATGKGAAASTQEGKSQPFKVTPGPFDPATWLEW<br>SRQWQGTEGNGHAAASGIPGLDALAGVKIAPAQLGDIQQRYMKDFSALWQAMAEGKAEATGPLHDRRFAG<br>DAWRTNLPYRFAAAFYLLNARALTELADAVEADAKTRQRIRFAISQWVDAMSPANFLATNPEAQRLLIESGGES<br>LRAGVRNMMEDLTRGKISQTDESAFEVGRNVAVTEGAVVFENEYFQLLQYKPLTDKVHARPLLMVPPCINKYYI<br>LDLQPESSLVRHVVEQGHTVFLVSWRNPDASMAGSTWDDYIEHAAIRAIEVARDISGQDKINVLGFCVGGTIVS<br>TALAVLAARGEHPAASVTLLTTLLDFADTGILDVFVDEGHVQLREATLGGGAGAPCALLRGLELANTFSFLRPND<br>LVWNYVVDNYLKGNTPVPFDLLFWNGDATNLPGPWYCWYLRHTYLQNELKVPGKLTVCGVPVDLASIDVPTYI<br>YGSREDHIVPWTAAYASTALLANKLRFVLGASGHIAGVINPPAKNKRSHWTNDALPESPQQWLAGAIEHHGS<br>WWPDWTAWLAGQAGAKRAAPANYGNARYRAIEPAPGRYVKAKA

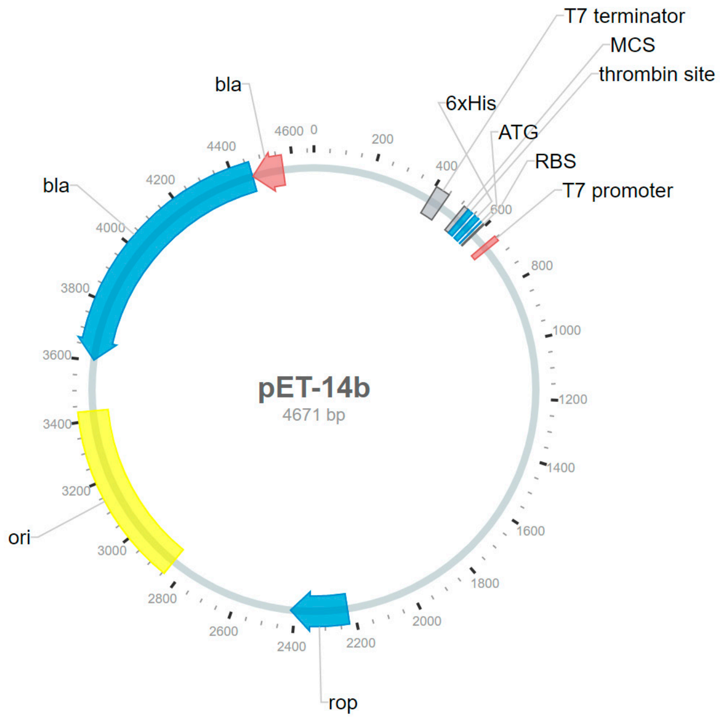

Figure S1: pET-14b vector used in this experiment.

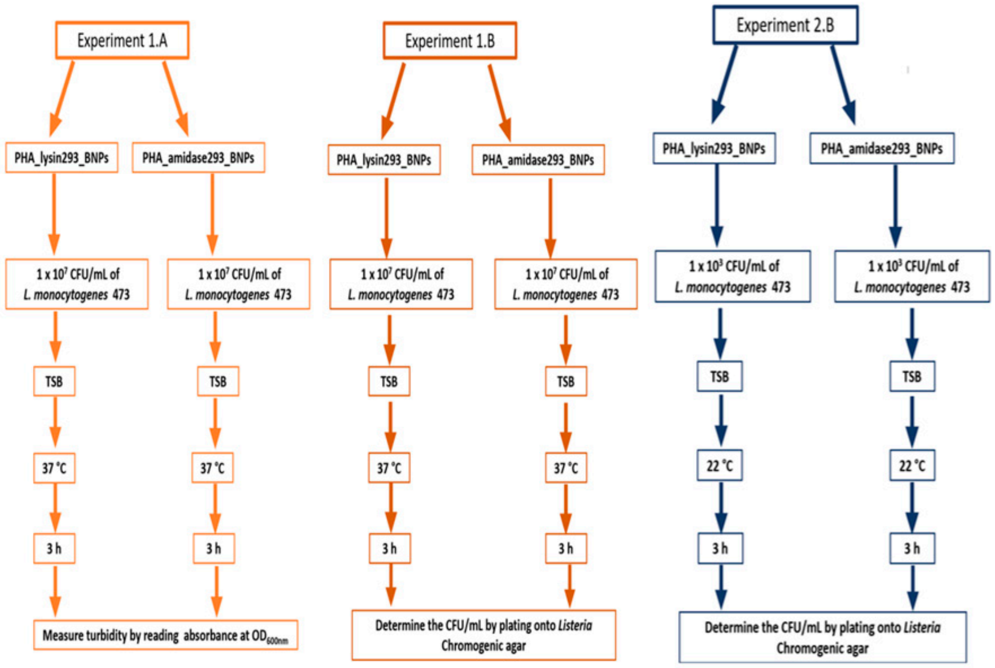

Figure S2: Flow chart depicting the experimental design of the assays. The flow chart shows the CFU/mL, temperature, incubation time, proteins used and the method to determine the CFU/mL or the turbidity of the samples in each experiment.
